# Atlas of Salivary Gland Tumor Cytopathology, Oral & Surgical Pathology – CD-ROM Disk 2: by Kini S. & Dardick I. Pathology Images Inc, 2006

**DOI:** 10.1186/1742-6413-3-26

**Published:** 2006-11-21

**Authors:** Zainab Basir

**Affiliations:** 1 Director of Cytology Laboratory, Associate Professor of Pathology, Department of Pathology, Medical College of Wisconsin, Milwaukee, USA

## Abstract

This CD-ROM version of Atlas of Salivary Gland Tumor Cytopathology, Oral & Surgical Pathology is an excellent and concise tool for easy reference during sign out of cytology and surgical cases. It is also invaluable as a teaching tool for residents and fellows.

Atlas of Salivary Gland Tumor Cytopathology, Oral & Surgical Pathology CD-ROM (ISBN 0-9736518-0-7), Published by Pathology images Inc Ottawa, Ontario K2B 7L4, Canada

## Review of CD

The Atlas of Salivary Gland Tumor Cytopathology, Oral & Surgical Pathology as a CD-ROM version is a very welcome edition to the library of any pathologist for quick reference [1]. The pathology of salivary gland tumors is so varied and the tumors are infrequently encountered in many pathology practices. Therefore, to have easy access with this CD-ROM to both cytologic features and surgical morphology in the same text is a very valuable tool for easy reference during sign out. It is also a useful tool for teaching residents and fellows.

The authors have organized this CD-ROM into convenient chapters. In the sections on specific tumors, the authors have nicely provided tables, brief cytologic and surgical text and excellent colored photographs with key features highlighted. Some photographs use arrows to illustrate the salient features. The majority of the photographs are of high quality, although an occasional one has been taken at too low power to fully discern the specific features.

I would recommend this CD-ROM in the cytology sign out room as a very useful quick reference for differential diagnosis of salivary gland tumors. The CD-ROM is easy to use, very concise in the information provided with high quality colored illustrations.

## Competing interests

The author(s) declare that they have no competing interests.

## Authors' contributions

The author critically reviewed the CD-ROM of Atlas of Salivary Gland Tumor Cytopathology, Oral & Surgical Pathology. Thereafter drafted and revised the manuscript of this review article.
